# Supplementary material for: A diversity of novel type-2 innate lymphoid cell subpopulations revealed during tumour expansion
Source: Commun Biol. 2024 Jan 3;7:12. doi: 10.1038/s42003-023-05536-0 (PMC10764766; doi:10.1038/s42003-023-05536-0)
Supplement: Supplementary file 2 — Supplementary Information [file 42003_2023_5536_MOESM2_ESM.pdf]

Supplementary Figures

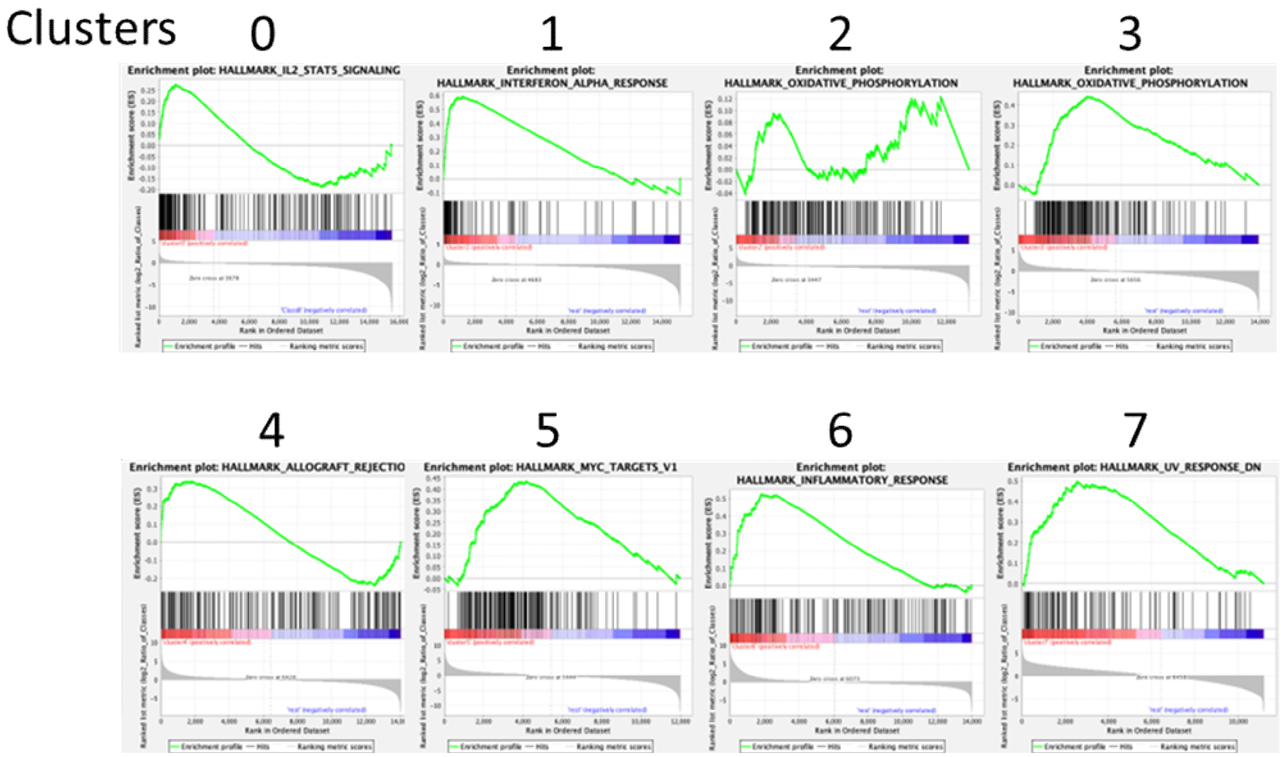

Supp Figure 1. Gene Set Enrichment Analysis (GSEA) of each cluster comparing to the rest of the whole ILC2 population. Mouse-ortholog hallmark gene sets are used as *priori* reference gene sets. Each enrichment plot is the upregulated hallmark pathway for the respective cluster.
