# Supplementary material for: A diversity of novel type-2 innate lymphoid cell subpopulations revealed during tumour expansion
Source: Commun Biol. 2024 Jan 3;7:12. doi: 10.1038/s42003-023-05536-0 (PMC10764766; doi:10.1038/s42003-023-05536-0)
Supplement: Supplementary file 3 — Reporting Summary [file 42003_2023_5536_MOESM3_ESM.pdf]

nature portfolio

Corresponding author(s): Dr Wilfred Jefferies

Last updated by author(s): Oct 21, 2023

Reporting Summary

Nature Portfolio wishes to improve the reproducibility of the work that we publish. This form provides structure for consistency and transparency in reporting. For further information on Nature Portfolio policies, see our Editorial Policies and the Editorial Policy Checklist.

Statistics

For all statistical analyses, confirm that the following items are present in the figure legend, table legend, main text, or Methods section.

n/a	Confirmed	
☐	☒	The exact sample size (*n*) for each experimental group/condition, given as a discrete number and unit of measurement
☐	☒	A statement on whether measurements were taken from distinct samples or whether the same sample was measured repeatedly
☐	☒	The statistical test(s) used AND whether they are one- or two-sided<br>*Only common tests should be described solely by name; describe more complex techniques in the Methods section.*
☐	☒	A description of all covariates tested
☐	☒	A description of any assumptions or corrections, such as tests of normality and adjustment for multiple comparisons
☐	☒	A full description of the statistical parameters including central tendency (e.g. means) or other basic estimates (e.g. regression coefficient) AND variation (e.g. standard deviation) or associated estimates of uncertainty (e.g. confidence intervals)
☒	☐	For null hypothesis testing, the test statistic (e.g. *F*, *t*, *r*) with confidence intervals, effect sizes, degrees of freedom and *P* value noted<br>*Give P values as exact values whenever suitable.*
☒	☐	For Bayesian analysis, information on the choice of priors and Markov chain Monte Carlo settings
☒	☐	For hierarchical and complex designs, identification of the appropriate level for tests and full reporting of outcomes
☒	☐	Estimates of effect sizes (e.g. Cohen's *d*, Pearson's *r*), indicating how they were calculated

Our web collection on statistics for biologists contains articles on many of the points above.

Software and code

Policy information about availability of computer code

Data collection	No Software was used for data collection
Data analysis	cellranger-6.0.0 was used for raw data processing and filtering.<br>Seurat V3 was used for downstream analysis. We followed the recommended settings of the standard workflow by Seurat.

For manuscripts utilizing custom algorithms or software that are central to the research but not yet described in published literature, software must be made available to editors and reviewers. We strongly encourage code deposition in a community repository (e.g. GitHub). See the Nature Portfolio guidelines for submitting code & software for further information.

Data

Policy information about availability of data

All manuscripts must include a data availability statement. This statement should provide the following information, where applicable:

- Accession codes, unique identifiers, or web links for publicly available datasets
- A description of any restrictions on data availability
- For clinical datasets or third party data, please ensure that the statement adheres to our policy

Sequencing and processed data are available and deposited at GEO, accession number GSE246502.

Research involving human participants, their data, or biological material

Policy information about studies with human participants or human data. See also policy information about sex, gender (identity/presentation), and sexual orientation and race, ethnicity and racism.

Reporting on sex and gender	N/A
Reporting on race, ethnicity, or other socially relevant groupings	N/A
Population characteristics	N/A
Recruitment	N/A
Ethics oversight	N/A

Note that full information on the approval of the study protocol must also be provided in the manuscript.

Field-specific reporting

Please select the one below that is the best fit for your research. If you are not sure, read the appropriate sections before making your selection.

☒ Life sciences ☐ Behavioural & social sciences ☐ Ecological, evolutionary & environmental sciences

For a reference copy of the document with all sections, see nature.com/documents/nr-reporting-summary-flat.pdf

Life sciences study design

All studies must disclose on these points even when the disclosure is negative.

Sample size	Samples were collected from pooled 5 mice per group. Samples are pooled to acquired enough cells for single cell sequencing. Gene expressions are calculated as log fold change based on single cell sequencing of 1500-4500 cells per sample. Stats are done by Wilcoxon Rank Sum test within Seurat package.
Data exclusions	No data exclusion.
Replication	Samples were collected at two time points, for each of the activated group and non-activated group. Samples of the same activation status are merged for downstream analysis.
Randomization	Randomization is no relevant in this study. Appropriate control is included for comparisons.
Blinding	Blinding was not relevant to the study. Experiments are carried out in parallel between groups, all groups received the same handling, not prone to objective bias.

Reporting for specific materials, systems and methods

We require information from authors about some types of materials, experimental systems and methods used in many studies. Here, indicate whether each material, system or method listed is relevant to your study. If you are not sure if a list item applies to your research, read the appropriate section before selecting a response.

Materials & experimental systems

n/a	Involved in the study	
☐	☒	Antibodies
☐	☒	Eukaryotic cell lines
☒	☐	Palaeontology and archaeology
☐	☒	Animals and other organisms
☒	☐	Clinical data
☒	☐	Dual use research of concern
☒	☐	Plants

Methods

n/a	Involved in the study	
☒	☐	ChIP-seq
☐	☒	Flow cytometry
☒	☐	MRI-based neuroimaging

Antibodies

Antibodies used	CD3ε/γ #11-0032-80, CD4 #11-0042-81, CD8α #11-0081-81, CD19 #11-0193-81, TCRβ #11-5961-81, NK1.1 #11-5941-81, TER119

Antibodies used	#11-5921-81, CD11c #11-0114-81, CD11b #11-0112-41 and Ly-6G/C #11-9668-80 (Thermo Fisher). IL-7 receptor, #135009 (Biolegend), IL-33 receptor, #145312(BIOLEGEND), BV605-conjugated Thy1.2 (#140317) and BV421-conjugated CD45 (#103133)
Validation	Validation described at manufacturer's website.

Eukaryotic cell lines

Policy information about cell lines and Sex and Gender in Research

Cell line source(s)	The TC1 cell line is a murine lung tumour model derived from primary lung epithelial cells of C57BL/6 mice immortalized using the amphotropic retrovirus vector LXSN16 carrying human papillomavirus (HPV) genes E6/E7, and subsequently transformed with pVEJB plasmid expressing the activated human c-Ha-ras
Authentication	None of the cell lines used were authenticated.
Mycoplasma contamination	All cell lines tested negative for mycoplasma contamination.
Commonly misidentified lines (See ICLAC register)	*Name any commonly misidentified cell lines used in the study and provide a rationale for their use.*

Animals and other research organisms

Policy information about studies involving animals; ARRIVE guidelines recommended for reporting animal research, and Sex and Gender in Research

Laboratory animals	C57Bl/6 Mus musculus
Wild animals	n/a
Reporting on sex	Female mice are used. Females are used because they generally yield higher amount of ILC2s, and ILC2s from female mice are more sensitive to activation signals.
Field-collected samples	n/a
Ethics oversight	This study was ethically approved by the Animal Care Committee (ACC) at the University of British Columbia (UBC).

Note that full information on the approval of the study protocol must also be provided in the manuscript.

Plants

Seed stocks	*Report on the source of all seed stocks or other plant material used. If applicable, state the seed stock centre and catalogue number. If plant specimens were collected from the field, describe the collection location, date and sampling procedures.*
Novel plant genotypes	*Describe the methods by which all novel plant genotypes were produced. This includes those generated by transgenic approaches, gene editing, chemical/radiation-based mutagenesis and hybridization. For transgenic lines, describe the transformation method, the number of independent lines analyzed and the generation upon which experiments were performed. For gene-edited lines, describe the editor used, the endogenous sequence targeted for editing, the targeting guide RNA sequence (if applicable) and how the editor was applied.*
Authentication	*Describe any authentication procedures for each seed stock used or novel genotype generated. Describe any experiments used to assess the effect of a mutation and, where applicable, how potential secondary effects (e.g. second site T-DNA insertions, mosiacism, off-target gene editing) were examined.*

Flow Cytometry

Plots

Confirm that:

- ☒ The axis labels state the marker and fluorochrome used (e.g. CD4-FITC).
- ☒ The axis scales are clearly visible. Include numbers along axes only for bottom left plot of group (a 'group' is an analysis of identical markers).
- ☒ All plots are contour plots with outliers or pseudocolor plots.
- ☒ A numerical value for number of cells or percentage (with statistics) is provided.

Methodology

Sample preparation	ILC2s derived from TC1 tumour-bearing mice (ta-ILC2s) were fixed and permeated using an intracellular fixation and permeabilization buffer set (eBioscience #88-8824-00) and then stained in PBS (2% FBS) with the following mouse antibodies: PE-conjugated granzyme B antibody (BioLegend #372207) and APC-conjugated granzyme C (BioLegend #150803). The IFITM1

	mouse monoclonal antibody was purchased from Proteintech (#60074-1-Ig) and conjugated with a FITC antibody labeling kit (Mix-n-Stain #92295).
Instrument	BD FACS Aria II
Software	Flow data anlyzed on Flowjo.
Cell population abundance	Refer to Figure 1.
Gating strategy	*Describe the gating strategy used for all relevant experiments, specifying the preliminary FSC/SSC gates of the starting cell population, indicating where boundaries between "positive" and "negative" staining cell populations are defined.*

☒ Tick this box to confirm that a figure exemplifying the gating strategy is provided in the Supplementary Information.
